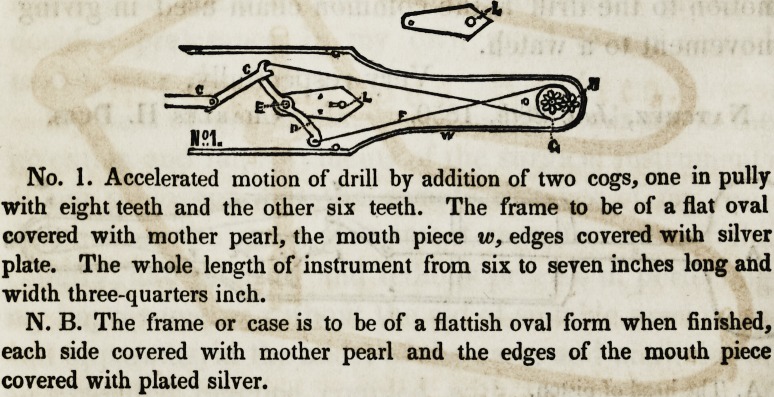# Dental Drills

**Published:** 1850-04

**Authors:** Charles H. Dubs

**Affiliations:** Natchez,


### ARTICLE VIII.

### DENTAL DRILLS.

*Messrs. Editors.*—Having seen in the Dental News Letter, for January, 1850, portrayed on the cover, the superficial figure of a new Dental Drill Stock, patented by Kerby Spencer, Dentist No. 176 Broadway, New York, together with notices in commendation of the same, I was struck with the external resemblance of the Spencer patent drill to an invention of my own, in which the propelling power was given by a piston rod, although I had two different modes of communicating the propelling power to the wheels which gave the rotary motion to the drill. As a matter of some interest perhaps, to amateurs in the improvement of the various instruments of Dental Surgery, I herewith send you three drawings illustrating my invention. I do not know any thing of the internal structure of the Spencer drill, as I never saw one, nor have I ever read the description or specification of the instrument, and it may be possible that one or the other of my modes may bear a similarity to the Spencer patent, in which case I shall be extremely unwilling that any instrument should be made from my models. No honorable Dental Surgeon would ever wish to infringe, in the least, upon a right secured by the patent laws of his country.

In my own dental operations I have used the drill propelled by the spring handles, compressed by the muscular motion of the hand; but there was an unsteady motion communicated to the drill by the contraction of the muscles, and there was frequently much difficulty in giving the desired angle to the drill hole in the various perforations. I therefore, studied out the models I now send to you; but have never before given them to the notice of the profession, because I felt assured the cost of making the instrument would be a serious objection to most dentists whatever might be the use of the instrument in Dental Surgery. I have ever prefered a light, delicate, strait drill, propelled by a twirling motion given to it by the thumb and fore-finger, to the spring forked handle drill, and am not altogether sure that for all cases the straight shaft drill so propelled would be preferable, if delicately and skill-fully used, to my more intricate and certainly vastly more expensive models. Such as they are, however, if they are no encroachment upon the Spencer drill, I freely give them to the dental profession; giving a decided preference in my own mind to my second model, over the first.

My bent of genius almost irresistibly leads me to the invention and improvements of the surgical instruments of our profession, but the remote distance of my residence from the great instrument manufactories of the country, and the slow and tedious process of perfecting my improvements without the aid of suitable machinery and apparatus, in the midst of the cares and anxieties of an extended practice, coupled with the instruction of students, all have discouraged me from prosecuting my improvements, as otherwise I would have most gladly done. But, above all have I been discouraged by harsh irritating, malicious and piratical treatment extended towards that most useful instrument of my invention, of which I am also the patentee, the "Compound Union Screw Forceps," which I am bold to say no Dental Surgeon who has ever used one would be deprived of for the sum of one hundred dollars. It was at first so obstinately denied that I was the original inventor in the improvement of these forceps, that I was forced to secure a patent, contrary to my original intention of giving the improvement gratuitously to the profession. The next move of the most outrageous malice was to proclaim that the invention was of no value, which has been proved to be most palpably false by the piracy committed upon my patent, although deficient and inferior in its structure by a noted instrument maker.

My drawings of the three subjoined models, are so plain that no description of their movements is necessary to any one who has the least idea of mechanical powers. The chain I use to communicate the final motion to the drill is the common chain used in giving movement to a watch.

Very respectfully,

NATCHEZ, *Jan.* 28*th*, 1850. CHARLES H. DUBS.

A. The head of piston.
B. do. do. do.
C.C. do. joints of crank, piston and lever.
D. do. lever.
E. do. axis of lever.
F.F. do. small chain attached to pulley and ends of lever.
G. do. pulley with groove to fit chain.
H. do. hole in centre of axis of pulley and cog wheel for drill.
J.J. do. small holes in ends of lever for rivets to secure chain.
L. do. moveable bar to slide and regulate gearing and top of same.
M. do. stationed bar with hole in centre for piston to slide steady.

A. The head of shaft or piston.
B. do. do. do.
C.C. do. cog at end of do.
D. do. pinion wheel secured in upper part of pully of six or eight teeth.
E. do. pivot of pinion wheel.
F.F. do. two plates to secure pinion wheel and pulley.
G.G. do. two oblong holes to admit pinion and do. to slide to tighten chain.
H. do. pulley with the hole in centre of axis to admit burr drill.
J. do. cross bar to steady shaft or piston.
K.K. do. endless chain attached to two wheels or pulleys.
L. do. spiral spring secured to bar J., for shoulder of piston B, to strike when forced down too hard to prevent jarring.
M. The frame to contain the movement.

No. 1. Accelerated motion of drill by addition of two cogs, one in pully with eight teeth and the other six teeth. The frame to be of a flat oval covered with mother pearl, the mouth piece *w*, edges covered with silver plate. The whole length of instrument from six to seven inches long and width three-quarters inch.

N. B. The frame or case is to be of a flattish oval form when finished, each side covered with mother pearl and the edges of the mouth piece covered with plated silver.

## Figures and Tables

**Figure f1:**
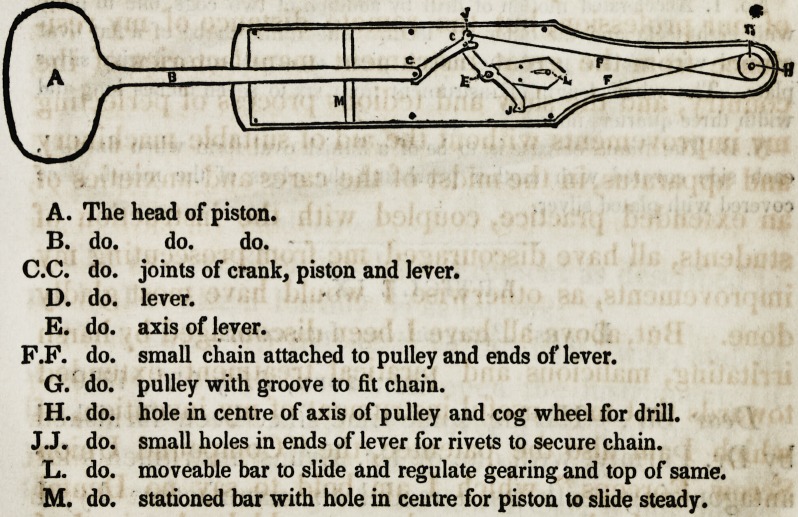


**Figure f2:**
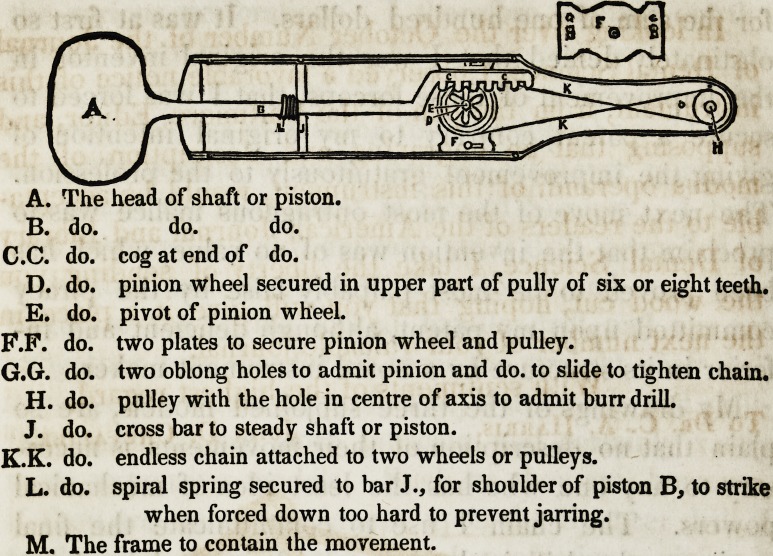


**Figure f3:**